# Pullulanase Is Necessary for the Efficient Intracellular Growth of *Francisella tularensis*

**DOI:** 10.1371/journal.pone.0159740

**Published:** 2016-07-22

**Authors:** Akihiko Uda, Neekun Sharma, Kazuhiro Takimoto, Tian Deyu, Yuuki Koyama, Eun-sil Park, Osamu Fujita, Akitoyo Hotta, Shigeru Morikawa

**Affiliations:** 1 Department of Veterinary Science, National Institute of Infectious Diseases, Shinjuku, Tokyo, Japan; 2 Division of Experimental Animal Research, National Institute of Infectious Diseases, Shinjuku, Tokyo, Japan; 3 Laboratory of Veterinary Public Health, Graduate School of Agricultural and Life Science, The University of Tokyo, Bunkyo, Tokyo, Japan; 4 United Graduate School of Veterinary Science, Gifu University, Gifu, Japan; University of Louisville, UNITED STATES

## Abstract

Pullulanase, an enzyme that catalyzes the hydrolysis of polysaccharides, has been identified in a broad range of organisms, including bacteria, yeasts, fungi, and animals. The pullulanase (*pulB*; *FTT_0412c*) of *F*. *tularensis* subspecies *tularensis* Schu S4 is considered to be a homologue of the type I pullulanase (*pulA*) of the other *Francisella* subspecies. The significance of *Francisella* pullulanase has been obscure until now. In the present study, we characterized a recombinant PulB of *F*. *tularensis* SCHU P9, which was expressed as a his-tagged protein in *Escherichia coli*. The recombinant PulB was confirmed to be a type I pullulanase by its enzymatic activity *in vitro*. A *pulB* gene knockout mutant of *F*. *tularensis* SCHU P9 (Δ*pulB*) was constructed using the TargeTron Knockout system and plasmid pKEK1140 to clarify the function of PulB during the growth of *F*. *tularensis* in macrophages. The intracellular growth of the Δ*pulB* mutant in murine macrophage J774.1 cells was significantly reduced compared with that of the parental strain SCHU P9. Expression of PulB in Δ*pulB*, using an expression plasmid, resulted in the complementation of the reduced growth in macrophages, suggesting that PulB is necessary for the efficient growth of *F*. *tularensis* in macrophages. To assess the role of PulB in virulence, the knockout and parent bacterial strains were used to infect C57BL/6J mice. Histopathological analyses showed that tissues from Δ*pulB*-infected mice showed milder lesions compared to those from SCHU P9-infected mice. However, all mice infected with SCHU P9 and Δ*pulB* showed the similar levels of bacterial loads in their tissues. The results suggest that PulB plays a significant role in bacterial growth within murine macrophage but does not contribute to bacterial virulence *in vivo*.

## Introduction

*Francisella tularensis*, the etiological agent of tularemia, is a gram-negative intracellular bacterium. *F*. *tularensis* poses a potential threat to both humans and animals as infection with only a few bacteria causes disease [1]. *F*. *tularensis* has been classified into three subspecies (*tularensis*, *holarctica*, and *mediasiatica*) based on their genomic sequence homology [2]. The subspecies *tularensis* (the type A biovar), which is predominantly found in North America and is more virulent for humans than the subspecies *holarctica* (the type B biovar) and *mediasiatica*, is often associated with lethal pulmonary infections [3].

The life cycle of *F*. *tularensis* has a close relationship with phagocytes, such as macrophages and dendritic cells, in the infected hosts. The bacteria captured by phagocytes in the infected hosts are efficiently engulfed, immediately escape into the cytosol, and proliferate in the cytoplasm [4]. Several phagocytic receptors that support an efficient entry of the bacterium into phagocytes have recently been identified, including the mannose receptor [5–7], complement receptor (CR) 3 (CD11b/CD18) [5–8], scavenger receptor A [9], and nucleolin [10]. The bacteria experience starvation of carbon source, amino acids, and nitrogen immediately after phagocytosis [11–15]. However, *F*. *tularensis* is able to quickly escape from phagosomes into the cytosol during bacterial replication [4, 16–18] because all components are synthesized from carbon source, amino acids, and nitrogen.

Pullulanases are present in a broad range of organisms, including bacteria, yeasts, fungi, and animals and are involved in the hydrolysis of polysaccharides [19–22]. The enzymes are widely used in the saccharification process for the commercial production of glucose (C_6_H_12_O_6_), maltose (C_12_H_22_O_11_; two α-1,4-linked glucose molecules), and maltotriose (C_18_H_32_O_16_; three α-1,4-linked glucose molecules). Pullulanases cleave the α-1,6 glucosidic bonds in pullulan, which is a linear polymer of maltotriose units joined by α-1,6 glucosidic bonds. Recently, five groups of pullulanase have been proposed based on their substrate specificities and reaction products [21, 23, 24]. Type I pullulanases hydrolyze the α-1,6 glucosidic linkages in pullulan and branched oligosaccharides to yield maltotriose and linear oligosaccharides, respectively [21]. Type II pullulanases cleave both α-1,4 and α-1,6 glucosidic linkages in various polysaccharides [21]. Reports describing other types of pullulanases are limited [21].

The complete genome sequence of *F*. *tularensis* Schu S4, reported by Larsson *et al*. [25], revealed that a pullulanase (*pulB*; *FTT_0412c*) highly similar to the type I pullulanase (*pulA*) of the other *Francisella* strains is encoded in the genomic DNA. *Francisella* PulB has not been characterized at all. In this study, we cloned, expressed, purified, and characterized (optimal pH and temperature) the pullulanase of *F*. *tularensis* subsp. *tularensis* SCHU P9. We then assessed its contribution to the intracellular growth of *F*. *tularensis* in a murine macrophage cell line and to pathogenicity *in vivo*.

## Materials and Methods

### Ethics statement

The experiments with animals were performed in strict accordance with the Animal Experimentation Guidelines of the National Institute of Infectious Diseases. The protocol was approved by the Institutional Animal Care and Use Committee of the National Institute of Infectious Diseases (Permit number: 113030).

### Bacterial strains

*F*. *tularensis* subsp. *tularensis* SCHU P5 and P9, which are attenuated and virulent strains, respectively [26], were routinely grown in Chamberlain defined medium (CDM) or on Eugon-chocolate supplemented with 8% defibrinated sheep blood. *Escherichia coli* were grown in Luria-Bertani medium or on Luria-Bertani agar plates. When necessary, the medium was supplemented with 50 μg/ml kanamycin or with 7.5 μg/ml chloramphenicol for *F*. *tularensis* and *E*. *coli*. All *in vitro* bacteriological procedures involving *F*. *tularensis* were carried out in a biosafety level 3 facility in accordance with the regulations of National Institute of Infectious Diseases (NIID), Japan.

### Production and purification of recombinant PulB

The *pulB* gene was cloned into pCold™ TF DNA plasmid (Takara, Shiga, Japan) to express recombinant PulB protein. The full open reading frame (ORF) of the *pulB* gene was amplified by PCR using the pulB-ORF-F and -R primer pair (Table 1). Similarly, a fragment of the pCold™ TF DNA plasmid was amplified using pCold-F and -R as the *pulB* primer pair (Table 1). PCR was performed using a GeneAmp PCR System 9700 (Perkin Elmer, Foster City, CA, USA) in a 50 μl reaction mixture containing 1× PrimeSTAR Max DNA Polymerase (Takara, Shiga, Japan), 0.5 μM primers, and 1 ng template DNA. Reaction conditions were as follows: 30 cycles of 98°C for 10 sec, 55°C for 15 sec, and 72°C for 1 min, followed by a final extension at 72°C for 7 min. The PCR products were purified using NucleoSpin Gel and PCR Clean-up columns (Machery-Nagel, Duren, Germany). The amplified *pulB* gene was ligated into the pCold™ TF DNA plasmid using the In-Fusion HD Cloning Kit (Takara, Shiga, Japan) in accordance with the manufacturer’s instructions. The resulting plasmid DNA was used to transform Competent High DH5α (Toyobo, Tokyo, Japan) according to the manufacturer's instructions, and the transformed *E*. *coli* cells were subsequently spread onto LB agar plates containing 50 μg/ml ampicillin. The plates were incubated overnight at 37°C, and colonies resistant to ampicillin were selected. A single colony was cultured in 50 ml of LB broth, and then the plasmid purified using NucleoBond PC 100 (Macherey-Nagel GmbH &Co.) was verified by determining its sequence.

**Table 1:** Primers used to produce recombinant PulB.

Prime name	5' -> 3' sequences
pulB-ORF-F *^1^	ATGCAAGCAACAAATCAAAATATATG
pulB-ORF-R *^1^	ACTGTGTATAATCATTAACGAATAAG
pCold-R for pulB *^1^ *^2^ *^3^	ATTTGTTGCTTGCATcactttgtgatt**CAT**GGTG
pCold-F for pulB *^1^ *^4^ *^5^	ATGATTATACACAGTcatcatcatcatcatcac**TAG**GTAATCTCTGCTTAAAAGCAC

*^1^ The underline indicates the overlapping sequence of pulB ORF for In-Fusion cloning.

*^2^ The lower case letters indicate the TEE site.

*^3^ The bold letters indicate the start codon.

*^4^ The lower case letters indicate the 6xHis site.

*^5^ The bold letters indicate the stop codon.

The plasmid was used to transform Zip Competent Cell BL21(DE3) cells (Biodynamics Laboratory, Tokyo, Japan). The transformed cells were cultured on LB plates supplemented with 50 μg/ml ampicillin at 37°C for 18 h. A single colony was cultured in 1 L of LB broth supplemented with 50 μg/ml ampicillin at 37°C until the optical density at 600 nm reached approximately 3.0. After the culture was kept at 16°C for 30 min, expression of recombinant PulB protein was induced immediately by the addition of 1 mM isopropyl-β-D-1-thiogalactopyranoside (IPTG, final concentration). The culture was incubated for a further 24 h at 16°C. The bacterial culture was centrifuged at 5000 *g* for 15 min at 4°C, and the resulting bacterial pellet was resuspended in 10 ml of sample buffer containing 20 mM phosphate buffer (pH 7.4), 1% Triton X-100, 20 mM imidazole, 500 mM NaCl, and a complete mini EDTA-free tablet (Roche). The bacterial suspension was sonicated for 30 min (10 s treatments were repeated at 10 sec intervals) at 4°C with a Bioruptor UCD-250 (Cosmo Bio, Tokyo, Japan). Cellular debris was removed by centrifugation at 15,000 *g* for 10 min at 4°C, and then the supernatants were filtered through a 0.22 μm bottle-top filter (Corning, NY, USA).

The recombinant C-terminally his-tagged PulB protein was purified using a 1 ml HisTrap HP column (GE healthcare, Piscataway, NJ) on an AKTA start system (GE healthcare). The column was equilibrated with 5 ml of 20 mM phosphate buffer (pH 7.4) containing 20 mM imidazole and 500 mM NaCl. After the sample was applied to the HisTrap HP, the column was washed with 20 ml of equilibration buffer. The bound protein was eluted with a 20 mM to 500 mM imidazole gradient from the wash buffer to HisTrap Elution Buffer [20 mM phosphate buffer (pH 7.4), 500 mM imidazole and 500 mM NaCl]. Three ml of the peak fraction (>100 mAU at 280 nm) were pooled manually. The purity of the recombinant PulB protein was checked by SDS-PAGE using a 5%–20% gradient polyacrylamide gel.

### Thin-layer chromatography

The hydrolysis products produced by the recombinant PulB protein were analyzed using thin-layer chromatography (TLC). The recombinant PulB (0.4 μM) was incubated with 0.25% pullulan (Sigma, St. Louis, MO), maltotriose (Nakarai tesque, Kyoto, Japan), maltose (Wako, Osaka, Japan), and glucose (Wako) in 50 mM sodium phosphate buffer (pH 6.2) at 37°C for 24 h. Five microliter aliquots of each reaction mixture were spotted onto a Silica gel 60 TLC plate with concentrating zone (Millipore, Darmstadt, Germany). The plate was developed with a 2-propanol/acetic acid/water (4:1:1) solvent system. The plates were dried and sprayed with orcinol-sulphuric acid reagent. The plate was heated at 120°C for 10 min.

### The optimal pH and temperature of PulB activity

The recombinant PulB (0.4 μM) was incubated with 0.25% pullulan (Sigma). The effect of pH on PulB activity was examined at 37°C for 12 h using 20 mM sodium phosphate buffers at different pHs. The effect of temperature on PulB activity was examined in 20 mM sodium phosphate buffer (pH 6.2) at various temperatures for 12 h. At the end of the incubation periods, all samples were heat inactivated at 94°C for 10 min. The negative control samples without PulB were similarly treated. The release of reducing groups from pullulan by PulB protein was measured by using the dinitrosalicylic acid (DNS) method. The 50 μl of reaction was added into 150 μl of DNS reagent and then incubated at 94°C for 10 min. The intensity of the color formed at 540 nm was then measured by using an iMark Microplate Absorbance Reader (Biorad).

### Construction of a Δ*pulB* mutant of *F*. *tularensis subsp*. *tularensis* SCHU P9

A *pulB* gene knockout mutant of SCHU P9 (Δ*pulB*) was constructed using a mutagenesis system based on the group II intron of the *ltrB* gene of *Lactobacillus lactis* designed to function in *F*. *tularensis* and plasmid pKEK1140 (GenBank accession number: EU499313), a kind gift from Dr. Karl E. Klose (South Texas Center for Emerging Infectious Diseases and Department of Biology, University of Texas, San Antonio). The IBS, EBS1d, and EBS2 primer sets used to synthesize PCR fragments were designed using the Sigma–Aldrich computer-based TargeTron algorithm design site (http://www.sigmagenosys.com/targetron/). The primers shown in Table 2 were synthesized by Eurofinsgenomics (Tokyo, Japan). The PCR product treated with *Xho*I (New England Biolabs, Beverly, MA, USA) and *Bsr*GI (New England Biolabs) restriction enzymes was ligated into the pKEK1140 plasmid digested with the same enzymes. After ligation, competent cells of *E*. *coli* DH5α strains (Competent high DH5α, Toyobo, Tokyo, Japan) were transformed with the above plasmid. Transformants were selected on LB agar plates containing 50 μg/ml kanamycin, and the plasmid DNAs were purified using NucleoBond PC 100 columns (Macherey-Nagel GmbH &Co., Doren, Germany). The resultant plasmids were referred to as pKEK(*pulB*).

**Table 2:** Primers used to construct *F*. *tularensis* SCHU P9 mutant strains.

Prime name	5' -> 3' sequences
Primers for construction of mutants	
EBS universal	CGAAATTAGAAACTTGCGTTCAGTAAAC
pulB-105IBS *^1^	AAAACTCGAGATAATTATCCTTAACTAACAGTATTGTGCGCCCAGATAGGGTG
pulB -105EBS1d *^2^	CAGATTGTACAAATGTGGTGATAACAGATAAGTCAGTATTAGTAACTTACCTTTCTTTGT
pulB -105EBS2	TGAACGCAAGTTTCTAATTTCGATTTTAGTTCGATAGAGGAAAGTGTCT
Primers for insertion check	
pulB -50s	TCACCCACCTTAATGCAACTC
pulB -132a	AAAACCATCAGCTTGGATGC
Primers for construction of complementation plasmid	
pulB -comp-F	CGGGCCTAGGTACAATAGAGATTTTATAGTTTATG
pulB -comp-R	CCGGGTCGACTAACTGTGTATAATCATTAACG
Primers for produce of recombinant PulB protein.	
pulB-ORF-F *^3^	**ATG**CAAGCAACAAATCAAAATATATG
pulB-ORF-R *^4^	**CTA**ACTGTGTATAATCATTAACGAATAAG
pCold-F for pulB *^4^*^5^	ATTATACACAGT**TAG**GTAATCTCTGCTTAAAAGCA
pCold-R for pulB *^3^*^5^	ATTTGTTGCTTG**CAT**GTGATGATGATGATGA

*^1^*Hind*III restriction site on IBS primer that designed by the Sigma–Aldrich computer-based Targetronalgorithm was replaced to *Xho*I restriction site (underline).

*^2^ The underline indicates the *Bsr*GI restriction site.

*^3^ The bold indicate the start codon of *pulB* gene.

*^4^ The bold indicate the stop codon of *pulB* gene.

*^5^ The underline indicates the overlapping sequence of pulB ORF for In-Fusion cloning.

To generate a Δ*pulB* strain, the virulent *F*. *tularensis* strain SCHU P9 was cultured at 37°C until the culture reached an optical density at 600 nm of approximately 0.8. Bacteria harvested by centrifugation at 12,000 *g* for 2 min were washed three times with 0.5 M sucrose and then suspended in 0.5 M sucrose. One microgram of each pKEK(*pulB*) plasmid were then electroporated into *F*. *tularensis* SCHU P9 cells using a Bio-Rad micropulser (Bio-Rad) at 2.5 kV. The transformed bacteria were rescued in CDM at 30°C for 1 h and then cultured on chocolate Eugon agar plates containing kanamycin at 30°C. Intracellular bacterial plasmids were removed by further incubation of each mutant strain on chocolate Eugon agar plates without antibiotics at 37°C.

Intron insertions were identified in Δ*pulB* by colony PCR. Genomic DNA was extracted using the Sepagene (Sanko Junyaku, Tokyo, Japan) kit according to the manufacturer’s instructions. PCR was performed with Blend taq (Toyobo, Tokyo, Japan) using a combination of gene-specific and intron-specific primers. The PCR conditions were as follows: initial pre-denaturation at 94°C for 2 min followed by 35 cycles of denaturation at 94°C for 30 sec, annealing at 50°C for 30 sec, and extension at 72°C for 2 min. Intron insertions were also verified with DNA sequencing.

### Complementation of the Δ*pulB* mutant with wild-type *pulB*

To complement the Δ*pulB* mutant, the pNVU1 expression plasmid [26], designated p(cont) in this study, was modified. The *pulB* gene was amplified from SCHU P9 genomic DNA using the pulB-ORF-F and -R primer pair (Table 2). The pNVU1 plasmid was also amplified using the pCold-F for pulB and pCold-R for pulB primer pair (Table 2). PCR was performed with a GeneAmp PCR System 9700 (Perkin Elmer, Foster City, CA, USA) in a 50 μl reaction mixture containing 1× PrimeSTAR Max DNA Polymerase (Takara, Shiga, Japan), 0.5 μM primers, and 1 ng template DNA. Reaction conditions were as follows: 30 cycles of 98°C for 10 sec, 55°C for 15 sec, and 72°C for 1 min and a final extension at 72°C for 7 min. The PCR products were purified using NucleoSpin Gel and PCR Clean-up (Machery-Nagel). The two amplicons, which contained a 15-bp overlap with each other, were connected using an In-Fusion HD Cloning Kit (Clontech Laboratories). The resulting plasmid DNA, designated p(pulB), was used to transform Competent High DH5α (Toyobo, Tokyo, Japan) cells according to the manufacturer's instructions. The transformed *E*. *coli* cells were subsequently spread onto LB agar plates containing 7.5 μg/ml chloramphenicol. The plasmid was replicated in *E*. *coli* DH5α and purified using NucleoBond PC 100 (Machery-Nagel). The Δ*pulB* mutant was transformed with p(pulB) plasmid by electroporation. The transformed bacteria were pre-cultured in CDM at 37°C for 1 h, and then selected on chocolate Eugon agar plates (Becton Dickinson) containing 7.5 μg/ml chloramphenicol at 37°C for 3 days. Bacterial stocks were prepared by cultivating the respective strains in CDM at 37°C for 24 h and stored in CDM containing 10% glycerol at −80°C until use.

### Intracellular growth of wild-type and Δ*pulB* mutant bacteria in macrophages

Cells from the murine macrophage line J774.1 were routinely propagated in RPMI 1640 medium (Wako, Osaka, Japan) supplemented with 10% fetal bovine serum (FBS, Invitrogen, Auckland, New Zealand). Four days prior to infection, J774.1 cells stimulated with phorbol-12-myristate 13-acetate (PMA, Sigma–Aldrich, St. Louis, MO, USA) (10 ng/ml) were seeded in the wells of a Multiwell Primaria 24-well plate (Falcon, Franklin Lakes, NJ, USA) at a concentration of 0.5 × 10^5^ cells per well and then incubated at 37°C for 2 days. After 2 days, the medium was replaced with RPMI 1640 medium supplemented with 10% FBS but without PMA, and the cells were further cultivated at 37°C for 2 more days. The cells were infected with SCHU P9 or its Δ*pulB* mutant at a multiplicity of infection (MOI) of 10. After the addition of bacteria, the cells were centrifuged at 1000 *g* for 10 min and then incubated at 37°C for 60 min. Cells were then washed three times with RPMI 1640 without serum and cultured again with fresh RPMI 1640 containing gentamicin (50 μg/ml) to kill extracellular bacteria. The cells were incubated for 60 min at 37°C. After 60 min, the cells were washed three times with RPMI 1640 medium lacking serum and lysed by the addition of 0.1% Triton X-100 in CDM for 1 min. Fresh RMPI 1640 without serum was added immediately after the 1 min lysis period. The lysed cells were processed to allow counting of the surviving intracellular bacteria. The number of colony forming units (CFU) of viable bacteria released from the cells was determined by plating serially diluted samples of the lysed cell preparation on Eugon-chocolate agar plates. The same protocol was repeated for counting the bacteria released from the cells at 24 h after infection. Statistical significances were performed by two-way ANOVA in GraphPad Prism 5 software (La Jolla, CA). All experiments for each strain were carried out in triplicate in a BSL-3 laboratory.

### Virulence of wild-type and Δ*pulB* mutant bacteria in mice

Four C57BL/6J mice in each group (seven- to twelve-week-old females; SLC, Inc. Shizuoka, Japan) were intranasally inoculated with 10^3^ CFU of SCHU P9 and Δ*pulB* mutant bacteria under anesthesia with 0.08 μg medetomidine hydrochloride (Domitor; Orion Diagnostica, Espoo, Finland), 25 μg midazolam (Dormicum; AstellasPharma, Tokyo, Japan), and 0.3 μg butorphanol tartrate (Stadol; Bristol-Myers Squibb Company, Tokyo, Japan). Clinical signs and body weight were recorded daily up to 6 days post infection (dpi). In case of extreme weight loss (>20%), mice were humanely sacrificed by isoflurane inhalation. All animal experiments were performed in a P3A facility.

### Histopathological analyses of mice infected with SCHU9 and Δ*pulB* mutant bacteria

Tissue samples from the mice infected with SCHU P9 and Δ*pulB* mutant bacteria were fixed in 10% phosphate-buffered formalin. Fixed tissues were embedded in paraffin, sectioned, and stained with hematoxylin and eosin (HE). Immunohistochemical (IHC) staining for the detection of the *Francisella tularensis* LPS was performed on paraffin-embedded sections using anti-*Francisella tularensis* LPS monoclonal antibody (clone T14, HyTest Ltd., Turku, Finland), N-Histofine Simple Stain MAX PO (M) (Nichirei, Tokyo, Japan), 3,3’-diaminobenzidine (DAB), and hematoxylin by Sapporo General Pathology Laboratory Co. Ltd. (Hokkaido, Japan).

## Results

### Characterization of PulB from *F*. *tularensis* subsp. *tularensis* SCHU P9

**Fig 1:**
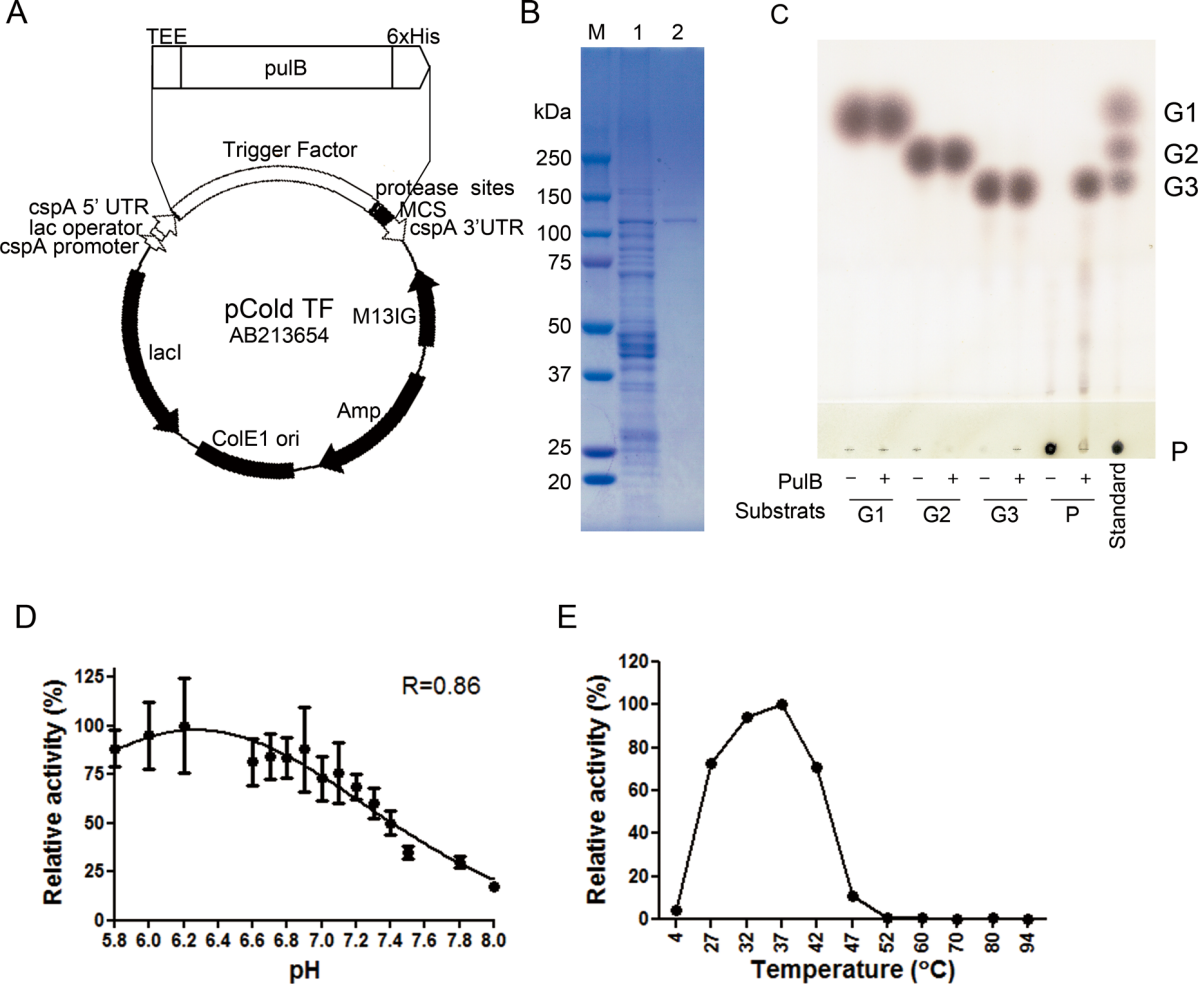
Characterization of the recombinant PulB of *F*. *tularensis* SCHU P9. (A) The plasmid pCold TF contains a *lac* operator, the cold shock protein A (*cspA*) 5' untranslated region (5' UTR), a translation enhancing element (TEE), a 6x His-tag sequence, the trigger factor sequence, protease cleavage sites, and a multiple cloning site (MCS) downstream of the *cspA* promoter. This plasmid, which is a cold shock expression vector, can express the target protein fused to a trigger factor under the control of the cold shock protein A (*cspA*) promoter and *lac* operator. In this study, the *pulB* gene fused with an N-terminal TEE sequence and a C-terminal 6x his-tag sequence was amplified from *F*. *tularensis* SCHU P9 DNA and then inserted into pCold TF plasmid lacking trigger factor, protease sites, and MCS using the In-Fusion HD Cloning Kit. (B) The expression and purification of the recombinant PulB. PulB expression was induced by IPTG in *E*. *coli* BL21(DE3) transformed with the pCold TF-pulB plasmid. Recombinant PulB was purified from the bacterial lysates using an AKTA start system equipped with a HisTrap HP column. Lane M, marker proteins (kDa); lane 1, *E*. *coli* BL21(DE3) lysates; lane 2, purified recombinant PulB. (C) The products of pullulan hydrolysis catalyzed by recombinant PulB are shown. Pullulan (P), maltotriose (G3), maltose (G2), and glucose (G1) were incubated in pH 6.2 phosphate buffer with (+) or without (–) the recombinant PulB at 37°C for 24 h. After this incubation, the samples were immediately heat denatured. The samples were subjected to TLC analysis using 2-propanol/acetic acid/water (4:1:1, vol/vol/vol) as the solvent system. (D and E) Effects of pH (D) and temperature (E) on recombinant PulB activity are shown. Recombinant PulB was incubated with 0.25% pullulan at 37°C for 12 h and then immediately heat denatured at 94°C for 15 min. The hydrolyzed products were measured in triplicate using the DNS method. After the background was subtracted from the data, the maximal relative activity was determined at pH 6.2 (D) and 37°C (E). Mean ± SD of relative activity are shown. The optimal pH was determined from a curve fitting (Gaussian) by GraphPad Prism software.

The sequence of the pullulanase (PulB; FTT_0412c; EC:3.2.1.41) of *F*. *tularensis* subsp. *tularensis* SCHU P9 was almost identical (identity = 0.992) with that of the pullulanase (FTN_0512) of *F*. *tularensis* subsp. *holarctica* LVS and those of the other species. Because the PulB of *F*. *tularensis* has not been characterized at all, we first examined the enzymatic activity of this PulB. The PulB of *F*. *tularensis* SCHU P9 with a C-terminal his-tag was expressed in *E*. *coli* BL21(DE3) harboring an expression plasmid based on the pCold TF DNA plasmid, from which the trigger factor, the HRV 3C protease site, the thrombin site, the Factor Xa site, and the multiple cloning (MCS) site were excluded (Fig 1A). *E*. *coli* BL21(DE3) harboring the plasmid was cultured at 37°C overnight and then exposed to 16°C for 24 h to produce the recombinant PulB using the *cspA* promoter. Recombinant PulB (predicted size = ∼123 kDa) was observed in the *E*. *coli* lysates using SDS-PAGE (Fig 1B, lane 1). After purification using an AKTA start system equipped with a HisTrap HP column, the purified recombinant PulB was obtained (Fig 1B lane 2).

To estimate the ability of *F*. *tularensis* PulB to hydrolyze pullulan, a mixture of pullulan and the purified PulB was incubated at 37°C for 24 h in phosphate buffer (pH 6.2). After incubation, the heat-inactivated sample was subjected to thin layer chromatography (TLC) (Fig 1C). Maltotriose (G3) was abundantly detected in the assay mixture, whereas neither glucose (G1) nor maltose (G2) was observed.

The enzymatic activity of the PulB was analyzed at various pHs and temperatures (Fig 1D and 1E). After PulB was incubated with 0.25% pullulan for 24 h, release of reducing groups from pullulan was measured in triplicate using the DNS method. The enzymatic reaction was found to proceed at its optimal rate at pH 6.2 and a temperature of 37°C.

### Construction of Δ*pulB* and the complemented strain

**Fig 2:**
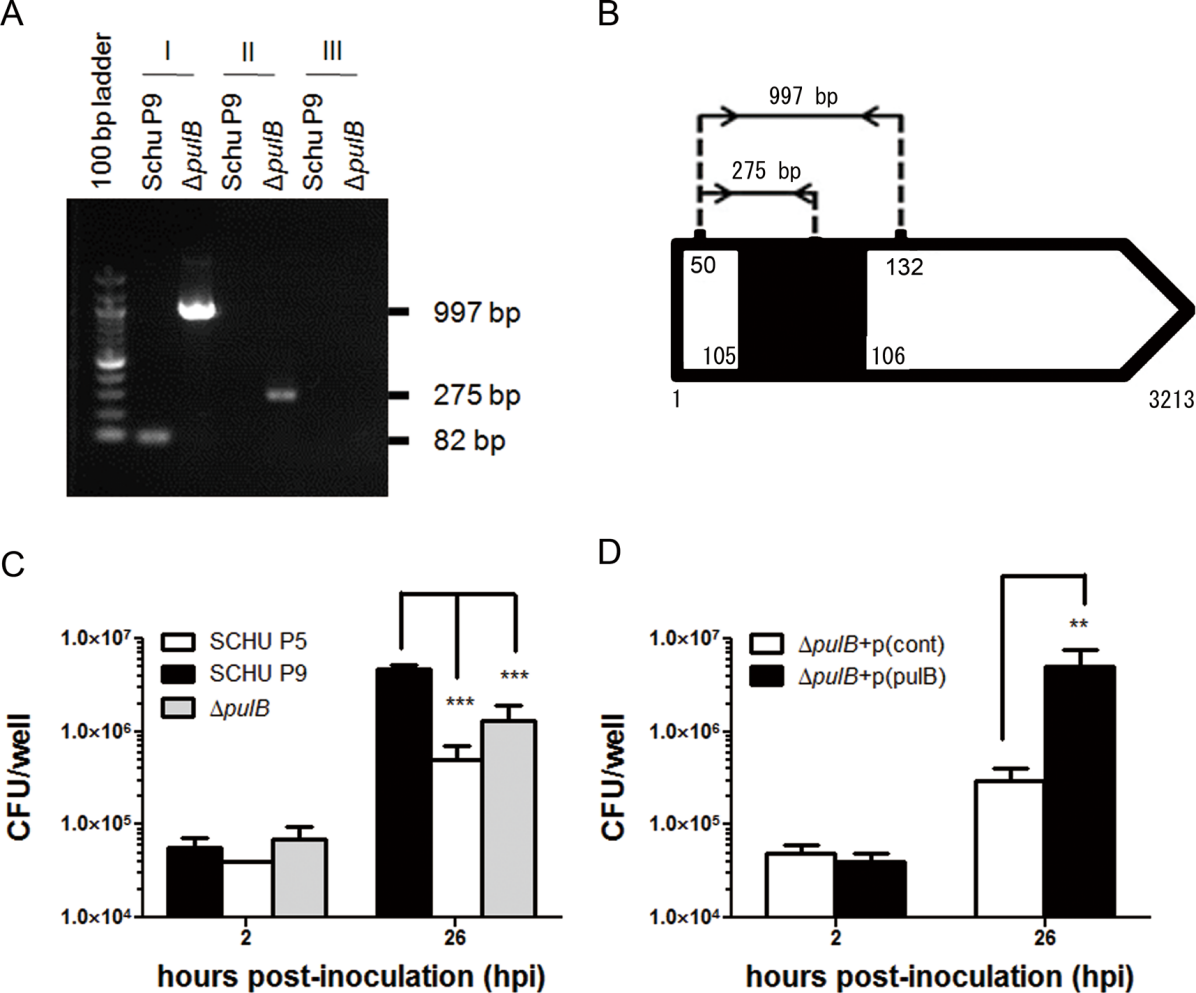
The intracellular growth of Δ*pulB* derived from virulent SCHU P9. (A) The insertion in Δ*pulB* derived from SCHU P9 was confirmed by PCR. Genomic DNA extracted from SCHU P9 and Δ*pulB* were subjected to PCR using gene specific sense/antisense (I), gene specific sense/EBS universal (II), and EBS universal/antisense (III) primer pairs. The amplicons and molecular weight marker were electrophoresed on a 0.7% agarose gel. (B) The schematic summary of Δ*pulB* generated in this study is shown. *ltr*B introns were inserted into the SCHU P9 *pulB* gene. The resultant mutant was designated Δ*pulB*. (C) J774.1 cells inoculated with virulent SCHU P9 (black bar), attenuated SCHU P5 (white bar), and Δ*pulB* (gray bar) at an MOI of 10, were incubated for 2 h and 26 h. Their intracellular CFUs were measured in triplicate. Mean ± SD of CFU are shown. Statistical significance was determined by using Student’s *t* test (***P < 0.001). (D) J774.1 cells were infected with Δ*pulB* complemented with a *pulB* gene expression plasmid (black bar) or a control plasmid (gray bar). The intracellular CFUs were measured in triplicate. Mean ± SD of CFU are shown. Statistical significance was determined by using Student’s *t* test (**P < 0.01).

A Δ*pulB* strain derived from SCHU P9 was produced using the TargeTron Knockout system and plasmid pKEK1140 as described by Stephen *et al* [27]. Considering the score on the Sigma–Aldrich computer-based TargeTron algorithm design site, an intron insertion position between nucleotides 105 and 106 of the *pulB* gene was selected. Subsequently, the intron re-targeting amplicons were produced using EBS universal, IBS, EBS1d, and EBS2 primers in each target (Table 2) and inserted into plasmid pKEK1140 to construct the plasmid pKEK(*pulB*). Bacteria transformed with pKEK(*pulB*) were cultured on chocolate Eugon agar plates containing kanamycin, and the insertion knockout mutants were checked by colony PCR (Fig 2A) using gene-specific and intron-specific primer sets of (Table 2). In Δ*pulB*, a 997 bp amplicon containing the insertion was detected using the FTT_0412c-50s and FTT_0412c-132a primer set, while a 275 bp DNA fragment was amplified using FTT_0412c-50s and EBS universal primers (Fig 2A). In contrast, no insertion was detected in the parent SCHU P9 strain. On the other hand, the polar effects of upstream (FTT_0413c; *glgB*) and downstream genes (FTT_0411c; *aroE2*) were not observed in Δ*pulB* (data not shown). These data demonstrate that the Δ*pulB* mutant was obtained as expected.

### Growth of Δ*pulB* in macrophages

To analyze the function of the *pulB* gene in bacterial virulence, intracellular growth of Δ*pulB* in murine macrophage J774.1 cells was compared with those of virulent SCHU P9 and attenuated SCHU P5 [26]. J774.1 cells inoculated with the bacteria at an MOI of 10 were cultured for 2 h and 26 h, and the number of intracellular bacteria was measured. As shown in Fig 2C, similar levels of bacteria were detected for all the three strains (Δ*pulB*, SCHU P5, and SCHU P9) within macrophages at 2 h post infection (hpi), indicating similar initial infection efficiency for all three strains. On the other hand, the CFU of SCHU P9 detected at 26 hpi was nine times higher than that of SCHU P5. Although the CFU of Δ*pulB* was intermediate between those of attenuated SCHU P5 and virulent SCHU P9, the CFU of Δ*pulB* at 26 hpi was significantly lower (*P* < 0.05, two-way ANOVA) than that of SCHU P9. With these data, it seems likely that the Δ*pulB* is phenotypically intermediate in bacterial virulence when compared with SCHU P5 and SCHU P9. Intracellular growth of Δ*pulB* was complemented with a *pulB* expressing plasmid, p(pulB), when compared with that of Δ*pulB* harboring the control plasmid, p(cont) (Fig 2D). Taken together, these data demonstrate that PulB plays a crucial role in the intracellular growth of *F*. *tularensis* SCHU P9 in murine macrophages.

### The pathogenicity of Δ*pulB* in mice

The pathogenicity of Δ*pulB* in mice was compared with that of the virulent SCHU P9 strain. Each group of four C57BL/6J mice (7-week-old females; SLC, Inc. Shizuoka, Japan) were intranasally inoculated with 10 μl containing 10^3^ CFU of bacteria under anesthesia with 0.08 μg medetomidine hydrochloride (Domitor; Orion Diagnostica, Espoo, Finland), 25 μg midazolam (Dormicum; AstellasPharma, Tokyo, Japan), and 0.3 μg butorphanol tartrate (Stadol; Bristol-Myers Squibb Company, Tokyo, Japan). The mice inoculated with Δ*pulB* showed rapid loss of up to 20% of their body weight and were sacrificed within 6 days.

**Fig 3:**
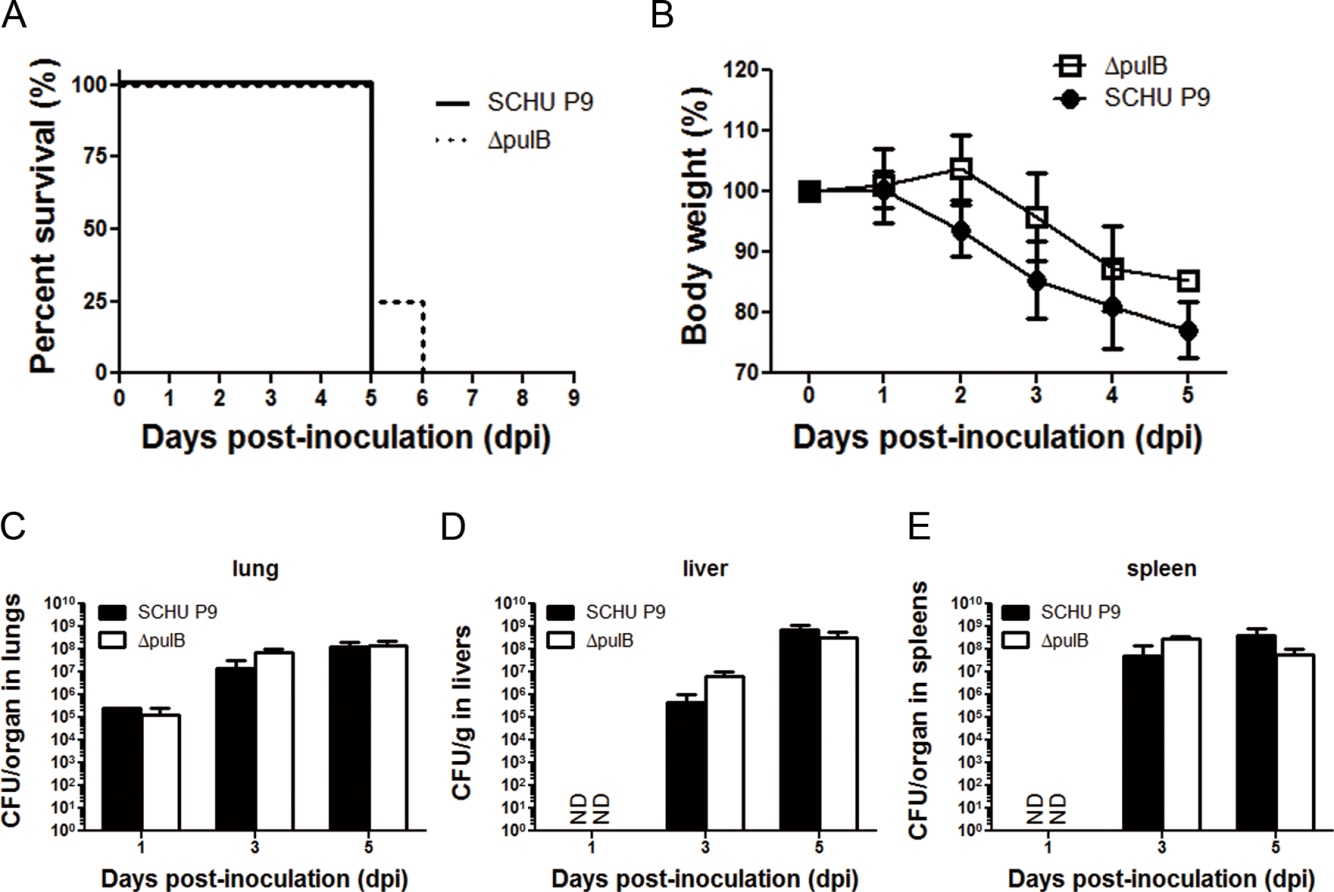
The comparisons of virulence of SCHU P9 and *ΔpulB* mutant bacteria in mice. Four C57BL/6J mice in each group (seven- to twelve-week-old females; SLC, Inc. Shizuoka, Japan) were intranasally inoculated with 10^3^ CFU of SCHU P9 and Δ*pulB*, respectively. Survival rates (A) and body weights (B) of these mice were measured up to 6 dpi. Mice were sacrificed at the indicated dpi, and then the averages ± SD of bacterial CFU in lungs (C), livers (D), and spleens (E) were shown. ND, not detected.

Onset of disease represented by body weight loss was delayed in the Δ*pulB*-infected mice (Fig 3A and 3B). However, the bacterial loads in lungs, livers, and spleens from the Δ*pulB*-infected mice were not significantly different from those from the SCHU P9-infected mice (Fig 3C, 3D and 3E).

**Fig 4:**
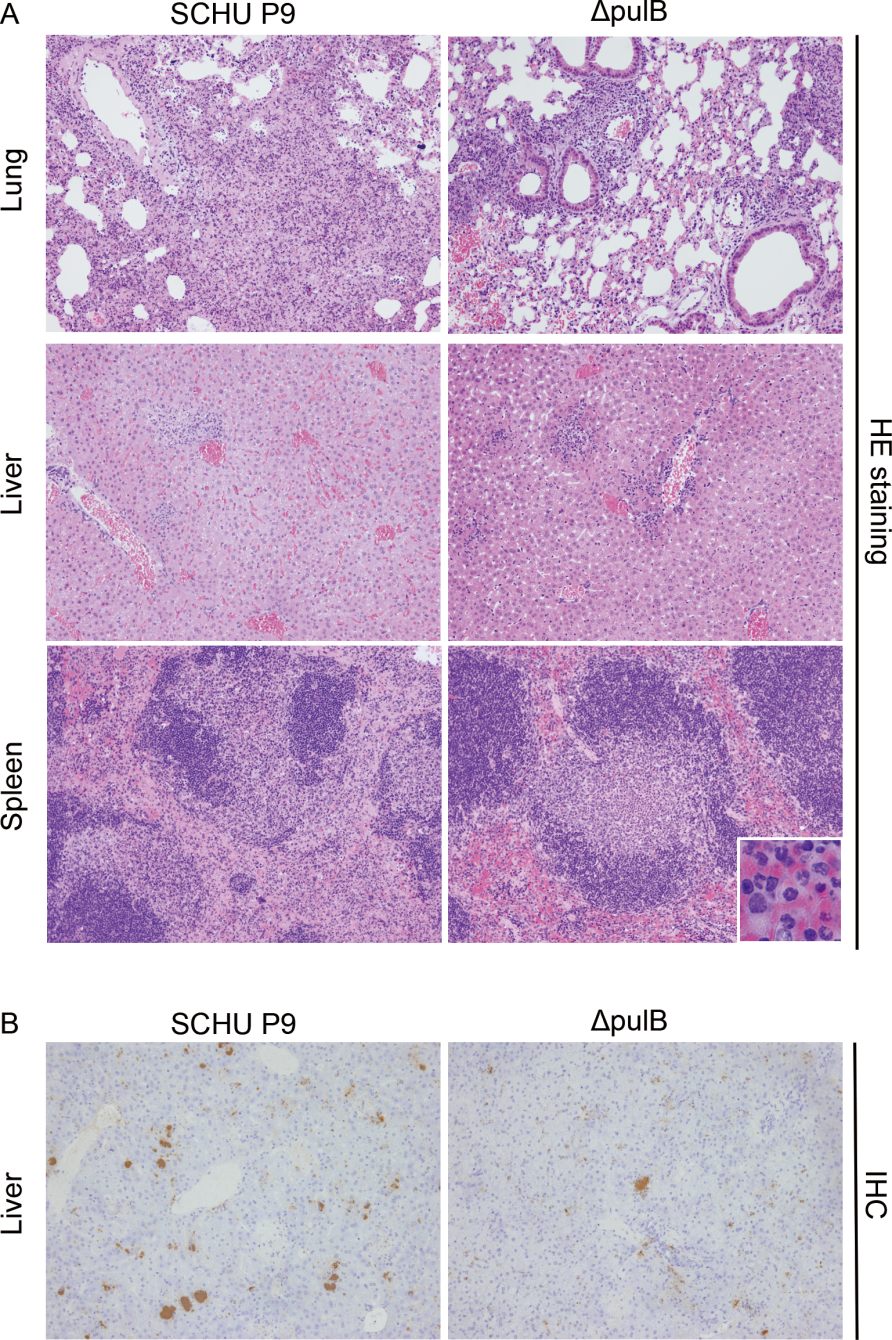
Histopathilogical analyses of mice infected with SCHU9 and Δ*pulB* mutant bacteria. Tissue sections obtained from mice infected with SCHU P9 and Δ*pulB* were examined by hematoxylin and eosin (HE) staining and immunohistochemistry (IHC) using anti-*Francisella tularensis* LPS monoclonal antibody. (A) Moderate focal necrosis and abscess were observed in lungs from mice infected with SCHU P9. Lungs from mice infected with Δ*pulB* showed milder pulmonary lesions compared to those from mice infected with SCHU P9. Vacuolar degeneration of hepatocytes, moderate focal necrosis and congestion were observed in livers of mice infected with SCHU P9. Livers from mice infected with Δ*pulB* showed milder focal necrosis. Marked focal necrosis associated with the accumulation of neutrophils was observed in the white pulp and red pulp of spleens from mice infected with SCHU P9 (3 dpi, original magnification x10). However, the accumulation of neutrophils was more prominent in the white pulp and around the central arteries or arterioles of spleens from mice infected with Δ*pulB* (right bottom square, original magnification x40). (B) Lung, liver and spleen from mice infected with SCHU P9 and Δ*pulB* were stained by immunohistochemical stain (IHC) with anti-*F*. *tularensis* LPS, N-Histofine Simple Stain MAX PO (M), and visualized by 3,3’-diaminobenzidine (DAB), followed by a hematoxylin counterstain (5 dpi, original magnification x10). LPS antigen positive foci, which are considered to be LPS-positive bacteria, were more prominent in SCHU P9-infected mice at 5 dpi compared to those in Δ*pulB*-infected mice.

Histopathological examination was performed to compare lesions caused by SCHU P9 and Δ*pulB*. Moderate to severe focal necrosis in parenchyma of lungs, livers and spleens was mainly observed in both SCHU P9- andΔ*pulB*-infected mice from 3 dpi although Δ*pulB*-infected mice had milder lesions than SCHU P9-infected mice (Fig 4A, S1 Fig and S1 Table). LPS antigen positive foci, which are considered to be LPS-positive bacteria, were more prominent in SCHU P9-infected mice at 5 dpi compared to those in Δ*pulB*-infected mice. (Fig 4B, S1 Fig and S1 Table). Neutrophils accumulated around the necrotic lesions or parenchyma in the lungs, livers and spleens from 3 dpi in both SCHU P9 and Δ*pulB*-infected mice. However, the accumulation of neutrophils was more prominent in the white pulp and around the central arteries or arterioles of spleens from mice infected with Δ*pulB* (Fig 4A).

Taken together, these results suggest that the *pulB* gene of *F*. *tularensis* plays an important role in bacterial growth within murine macrophage J774.1 cells, but it does not contribute to bacterial virulence *in vivo*.

## Discussion

The ability of *F*. *tularensis* to infect a wide variety of hosts may suggest an ability to adapt to diverse growth environments. Over the past few years, many researchers have focused on understanding the molecular and genetic bases of *F*. *tularensis* pathogenesis [28]. Although the intracellular fate of the bacterium has already been characterized [29], very little is known about the various virulence factors encoded in this organism, despite its extreme virulence. Many genes encoded within the *Francisella* pathogenicity island (FPI) have been shown to contribute to *F*. *tularensis* pathogenesis. However, knowledge of the functions and contributions of genes outside the FPI is relatively scarce. In this study, we focused on pullulanase (*pulB*; *FTT_0412c*).

When the isoelectric points (*p*I) and molecular weights (Mr) of the PulB were calculated *in silico* using DNASIS pro software (Hitachi software, Tokyo, Japan), the PulB of *F*. *tularensis* SCHU P9 was predicted to be a relatively large (Mr = 122,398), weakly acidic (*p*I = 6.53) protein. PROSITE [30], the bacterial localization prediction tool, predicted that PulB is localized to the cytoplasm of infected cells. The result of a BLAST search [(Algorithm blastp (protein-protein BLAST)] indicated that half of the C-terminal segment of PulB exhibits high sequence homology with type I pullulanase (TIGR02104: pulA_typeI). In addition, recombinant PulB hydrolyzed the α-1,6 glucosidic bonds in pullulan, and maltotriose was obtained as a final product in this study (Fig 1C). Taken together, these data strongly suggest that the PulB of *F*. *tularensis* SCHU P9 is a type I pullulanase.

Type 2 secretion systems (T2SSs), which translocate proteins from the periplasm through the outer membrane into the extracellular environment, have been identified in various bacteria, including *E*. *coli* [31], *Klebsiella* spp. [32, 33], *Vibrio cholera* [34], *Yersinia enterocolitica* [35], *Pseudomonas aeruginosa* [36, 37], and *Legionella pneumophila* [38]. In *K*. *pneumonia*, pullulanase is the only known folded protein secreted by the T2SS [39]. Recently, Tomas *et al*. reported that the pullulanase (PulA) knockout mutant of *K*. *pneumoniae*, which perturbed Toll-like receptor (TLR)-dependent recognition, is attenuated in the CD-1 mouse pneumonia model [40]. This evidence suggests that the pullulanase of *K*. *pneumoniae* is essential for full effectiveness in escaping the immune responses. [40]. These reports attracted our attention regarding *F*. *tularensis* pullulanase as a virulence factor.

To assess the role of pullulanase in *F*. *tularensis* pathogenicity, a *pulB* gene knockout mutant of *F*. *tularensis* SCHU P9 was constructed using the TargeTron Knockout system and plasmid pKEK1140 (Fig 2A and 2B). The *F*. *tularensis* SCHU P9 *pulB* gene was found to be associated with intracellular growth of the bacterium, since Δ*pulB* showed significantly less growth than SCHU P9 in macrophages (Fig 2C). This defect in Δ*pulB* was completely rescued by a *pulB* expression plasmid (Fig 2D). However, in contrast to these *in vitro* findings, mice infected with 1 × 10^3^ CFU of Δ*pulB* or SCHU P9 showed rapid loss of up to 20% of their body weight and had to be sacrificed within 6 days, even though onset of disease represented by body weight loss was delayed by 1 day in the Δ*pulB*-infected mice (Fig 3A and 3B). Histopathologically, lesions observed in various tissues from Δ*pulB*-infected mice were milder than those from SCHU P9-infected mice (Fig 4). However, in contrast, body weight loss and clinical signs were undetectable over a period of three weeks in mice infected with attenuated SCHU P5 in a previous study [26]. We also compared the levels of *pulB* mRNA expression in the attenuated SCHU P5 and virulent SCHU P9 strains using real-time RT-PCR with taqman probe (data not shown). The *pulB* expression level was slightly higher in virulent SCHU P9 than in attenuated SCHU P5 (fold change, 1.12; *P* = 0.004). These data strongly support the conclusion that *F*. *tularensis* SCHU P9 PulB makes some contribution to bacterial growth in macrophages, although it does not contribute to bacterial virulence *in vivo*. This might indicate that some other factors are necessary for the further attenuation of Δ*pulB in vivo*.

## Supporting Information

S1 Fig The time course of the pathologic changes in mice infected with SCHU9 and Δ*pulB* mutant bacteria. Tissue sections obtained from mice infected with SCHU P9 and Δ*pulB* at 1, 3 and 5 dpi were examined by hematoxylin and eosin (HE) staining and immunohistochemistry (IHC) using anti-*Francisella tularensis* LPS monoclonal antibody. Moderate focal necrosis and abscess were observed in lungs from mice infected with SCHU P9 at 3 dpi, while the lesions became severe at 5 dpi. Lungs from mice infected with Δ*pulB* showed milder pulmonary lesions compared to those from mice infected with SCHU P9 at 3 and 5 dpi. Vacuolar degeneration of hepatocytes, moderate focal necrosis and congestion were observed in livers of mice infected with SCHU P9 at 3 dpi, while the lesions became severe and many LPS-positive foci were appeared at 5 dpi. Livers from mice infected with Δ*pulB* showed milder focal necrosis at 3 and 5 dpi. Marked focal necrosis associated with the accumulation of neutrophils was observed in the white pulp and red pulp of spleens from mice infected with SCHU P9 at 3 dpi, while severe necrosis was observed at 5 dpi. The lesions were milder in spleens from mice infected with Δ*pulB*, however, the accumulation of neutrophils was more prominent in the white pulp and around the central arteries or arterioles of spleens from mice infected with Δ*pulB* at 3dpi. (Tissue sections with HE staining at 3dpi and IHC at 5dpi were also shown in Fig 4. Original magnification x10).
(TIF)

S1 Table Histopathological findings between mice infected with SCHU P9 and ΔpulB. (XLS)
