# Supplementary figures and images for: Pullulanase Is Necessary for the Efficient Intracellular Growth of Francisella tularensis
Source: PLoS One. 2016 Jul 22;11(7):e0159740. doi: 10.1371/journal.pone.0159740 (PMC4957787; doi:10.1371/journal.pone.0159740)

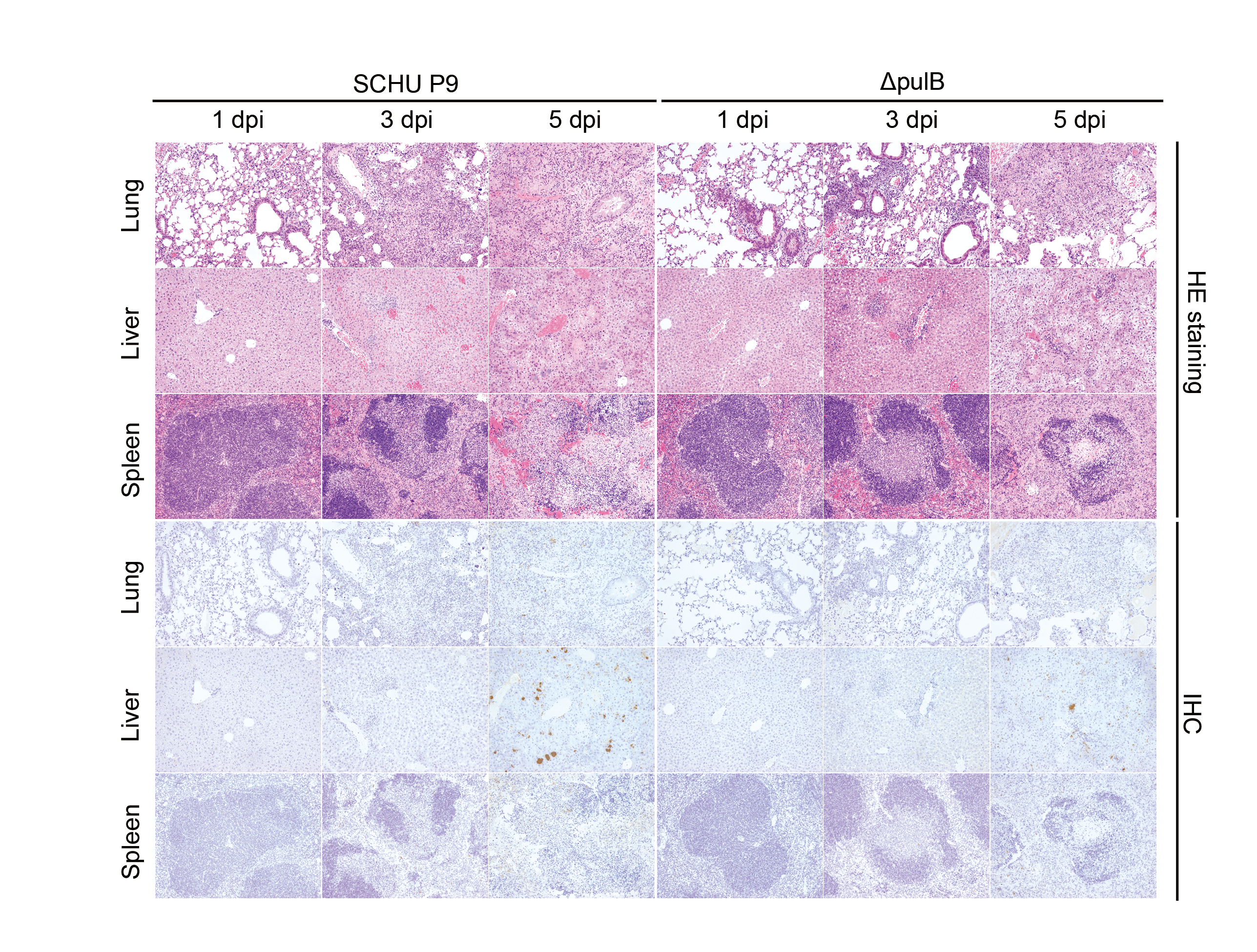

Supplement: S1 Fig — Tissue sections obtained from mice infected with SCHU P9 and ΔpulB at 1, 3 and 5 dpi were examined by hematoxylin and eosin (HE) staining and immunohistochemistry (IHC) using anti-Francisella tularensis LPS monoclonal antibody. Moderate focal necrosis and abscess were observed in lungs from mice infected with SCHU P9 at 3 dpi, while the lesions became severe at 5 dpi. Lungs from mice infected with ΔpulB showed milder pulmonary lesions compared to those from mice infected with SCHU P9 at 3 and 5 dpi. Vacuolar degeneration of hepatocytes, moderate focal necrosis and congestion were observed in livers of mice infected with SCHU P9 at 3 dpi, while the lesions became severe and many LPS-positive foci were appeared at 5 dpi. Livers from mice infected with ΔpulB showed milder focal necrosis at 3 and 5 dpi. Marked focal necrosis associated with the accumulation of neutrophils was observed in the white pulp and red pulp of spleens from mice infected with SCHU P9 at 3 dpi, while severe necrosis was observed at 5 dpi. The lesions were milder in spleens from mice infected with ΔpulB, however, the accumulation of neutrophils was more prominent in the white pulp and around the central arteries or arterioles of spleens from mice infected with ΔpulB at 3dpi. (Tissue sections with HE staining at 3dpi and IHC at 5dpi were also shown in Fig 4. Original magnification x10). (TIF) [file pone.0159740.s001.tif]
